# Supplementary material for: Freshwater Management Discourses in the Northern Peruvian Andes: The Watershed-Scale Complexity for Integrating Mining, Rural, and Urban Stakeholders
Source: Int J Environ Res Public Health. 2023 Mar 7;20(6):4682. doi: 10.3390/ijerph20064682 (PMC10048230; doi:10.3390/ijerph20064682)
Supplement: Supplementary file 1 [file ijerph-20-04682-s001.zip › S2.pdf]

INSTRUCCIONES PARA CONDUCIR ENTREVISTAS AL POBLADOR DE LA CUENCA DEL RIO MASHCÓN, CAJAMARCA-2018.

Antes de conducir la entrevista, el entrevistador debe asegurarse de tener a la mano los materiales necesarios: grabadora de voz, cuaderno de apuntes, GPS y una copia del protocolo por cada entrevistado. Se recomienda utilizar el lenguaje más sencillo posible durante la entrevista, así como una actitud respetuosa hacia el entrevistado.

Es importante evitar influenciar o dirigir la opinión del entrevistado. Es decir, **el entrevistador debe mantener una posición neutral sobre el tema investigado en todo momento**. Este aspecto es propio del enfoque interpretativo-constructivista de la presente investigación social. De preferencia, la entrevista se lleva a cabo previo acuerdo o cita con la persona a entrevistar o en otro caso se debe confirmar in-situ la disposición de la persona para ser entrevistada. El entrevistador debe considerar los siguientes pasos para abordar la entrevista:

Paso 1.- El entrevistador debe crear un **ambiente amigable e informal** desde la primera interacción con la persona a ser entrevistada cara a cara. Primero se debe expresar el **agradecimiento** a la persona por brindar unos minutos de su valioso tiempo.

Paso 2.- Seguidamente, el entrevistador hace una pequeña presentación de su persona, poniendo particular énfasis en la explicación de la razón por la que necesita conducir estas entrevistas (i.e. parte de una investigación académica). En este punto se indica el **objetivo general** de la entrevista: **"Recoger su opinión, percepción y conocimiento sobre los servicios que brindan los ríos y manantiales de la cuenca del río Mashcón, en Cajamarca".** Para brindar mayor detalle del objetivo, se puede dar algunos ejemplos de preguntas que contiene la entrevista.

Paso 3.- Se debe comunicar al entrevistado que la presente entrevista puede durar alrededor de 30 minutos, con flexibilidad de tiempo en caso la persona quisiera extenderse o no en sus respuestas. También, es importante comunicar que las preguntas serán abiertas. Es decir, **no existe respuesta correcta ni respuesta incorrecta**, sino que se desea solamente recoger opiniones sinceras. Es importante **garantizar la anonimidad del entrevistado o entrevistada**, comprometiéndose a que sus datos personales no serán publicados, y que los resultados de todas las entrevistas serán generalizados, sin indicar "quién" respondió "qué" en el reporte final. Es decir, se garantiza el manejo ético y confidencial de la información recogida. Adicionalmente se debe consultar a la persona si puede permitir que la entrevista sea grabada.

Paso 4.- Confirmada la predisposición de la persona, **se procede a dar inicio a la entrevista, a la grabación de la misma y al tomado de notas**. Primero se recogen datos personales del entrevistado como lugar de residencia, nombre, edad, sexo y ocupación. Para ello se debe mantener un ambiente de conversación casual. Por ejemplo, para obtener respuestas sobre el estado ocupacional y nivel de instrucción, se podría preguntar de manera casual: ¿A qué se dedica usted normalmente? y ¿Hasta qué nivel ha completado sus estudios?, respectivamente.

Paso 5: "La entrevista". - Una vez recogidos los datos generales del entrevistado, se procede a **realizar las preguntas semi-estructuradas**. En lo posible, se debe obtener respuestas para todas las preguntas del cuestionario. Las preguntas se realizan abiertamente, con el fin de mantener el ambiente de conversación casual que permita al

entrevistado o entrevistada explayarse al máximo. Por ello, en lugar de seguir un orden lineal, las preguntas han sido organizadas en cuatro grupos que se muestran en la tabla 1.

Tabla 1. Tipos de preguntas en entrevistas semi-estructuradas*

	Tipo	Descripción
Grupo 1 (G1)	Preguntas espontáneas	Son preguntas muy generales, sencillas y fundamentales que sirven para romper el hielo. Se usan para poner en una situación confortable al entrevistado, o también para cambiar a otro tema en medio de la entrevista.
Grupo 2 (G2)	Preguntas esenciales	Son aquellas sobre las cuales se ha construido la investigación. Es decir, son preguntas que abordan directamente la interrogante de investigación.
Grupo 3 (G3)	Preguntas adicionales	Consiste en un “parafraseo” de las preguntas esenciales, y sirven tanto para verificar la validez de la entrevista, así como para ayudar al entrevistado en caso no haya entendido completamente alguna pregunta del Grupo 2.
Grupo 4 (G4)	Preguntas indagatorias	Son aquellas preguntas que permiten al entrevistado elaborar sus respuestas. Buscan recoger las opiniones y sentimientos personales, promoviendo el pensamiento crítico en la persona entrevistada. Las respuestas pueden ser **exploradas más profundamente** con la ayuda de preguntas tipo ¿cómo?, ¿cuándo?, ¿por qué? y similares.

*Adaptado de L. Luo & B. M. Wildemuth (2009) Semi-structured interviews.

Para aplicar efectivamente los diferentes grupos de preguntas, se debe considerar la siguiente secuencia:

a) La conversación debe iniciar con preguntas del Grupo 1 (G1), a manera de ejercicio de calentamiento para afianzar al entrevistado. Las preguntas G1 también sirven en adelante para cualquier momento que se necesite desestresar la conversación.
b) Luego, a manera de seguir con la conversación, se deben realizar las preguntas esenciales del Grupo 2 (G2). Es importante que **el entrevistador no haga ningún tipo de comentario, ni demuestre emociones, en torno a las respuestas.**
c) Seguidamente, las preguntas del Grupo 3 (G3) buscan mayor detalle y corroboran las respuestas a las preguntas del G2. **Su enunciado es flexible**, sujeto a la necesidad de completar y/o verificar información de las preguntas del G2.
d) Las preguntas del Grupo 4 (G4) requieren del mayor tiempo, por lo que toman su lugar una vez que el entrevistado este más afianzado con el desarrollo de la entrevista. Estas preguntas recogen la mayor cantidad de información.
e) Finalmente, se verifica que se hayan cubierto todos los elementos del “checklist”, y de ser necesario se recoge la información faltante del entrevistado antes de cerrar y concluir con la entrevista.

ENTREVISTA PARA PERSONAS QUE HAN VIVIDO AL MENOS LOS ULTIMOS 5 AÑOS EN LA CUENCA DEL RIO MASHCÓN, CAJAMARCA-2018.

Lugar: …………………………………… hora y fecha: ……………………………

Nombre: …………………………………………Estudios completados:……………

Ocupación: ………………………………………Años… … Sexo………………….

Número de personas que viven en la misma casa…………………………………….

Años viviendo en la zona: …………… recursos económicos: ……………………...

Nombre de la fuente de agua, quebrada o río más cercanos: …………………………

Coordenadas GPS, X,Y, altitud:…………………………………………………….....

1. ¿Cómo empieza un día normal para usted? ¿Cómo es su rutina diaria? (G1)

2. ¿Utiliza su tiempo libre para ir a la ciudad?, Si lo hace ¿Con que frecuencia va usted a la ciudad? (G2)

3. ¿Y porque va? ¿O porque no va? (G3)

4. ¿Podría decirme si usted disfruta de dichas actividades que realiza (pregunta 3) durante esas visitas? ¿O quizás algo que le desagrade o incomode? (G4)

5. ¿Sabe de dónde proviene el agua que usamos en Cajamarca? ¿Y el agua que usa usted en su casa? (G2)

6. ¿Conoce el nombre de algunos ríos o quebradas, que usted considere importantes para la disponibilidad de agua en su hogar? (G3)

7. ¿Tiene alguna historia personal o anécdota en ese río/quebrada que desee compartir? (G4)

8. Personalmente, para usted, ¿Es mejor tener un día soleado y con calor? ¿O prefiere los días lluviosos, o nublados? (G1)

9. En su vivienda, ¿cuenta con sistema de agua <u>potable</u>? ¿y con agua de <u>canal</u> de irrigación? (G2)

10. ¿Alguna vez has percibido escasez de agua? (G2)

11. Sí respondió sí: ¿Cuándo? y ¿cuán seguido sucede? (G3)

12. ¿Cuál cree usted que sea la causa (de la respuesta a la pregunta 11)? (G4)

13. ¿Algunas veces tiene que comprar agua adicional para cubrir sus necesidades? (G3)

14. Sí es así, ¿de dónde la obtiene y cuánto le cuesta? (G3)

15. ¿En cuál de sus actividades diarias se utiliza la mayor cantidad de agua? (agricultura, ganadería, aseo personal, lavado de vehículos) (G1)

16. ¿Podría hacer memoria usted, si en el pasado se disponía de mayor o de menor cantidad de agua en su comunidad o vecindario? (G3)

17. ¿A qué cree que se deba este cambio (la respuesta a la pregunta 15)? (G4)

18. ¿Utiliza usted el agua del río? ¿Por qué? (G2-G3)

19. ¿Le sirve de algo a usted las orillas o riberas de los ríos? (ej.: para extraer piedras, para pastar el ganado, o para sentarse a descansar, o caminar) (G3)

20. ¿Sabe usted algo más de porqué son importantes las riberas (orillas o costados) de los ríos?

21. ¿Bebería usted el agua del río? ¿Por qué sí o por qué no? (G3)

22. ¿Bebería usted el agua que llega a su grifo? ¿Por qué sí o por qué no? (G3)

23. ¿Cómo se cerciora, o que medidas toma usted para que su agua que bebe sea saludable? ¿Aplica algún tratamiento, hervido, lejía, filtración? (G4)

24. ¿Alguna vez se ha enfermado, o sentido afectado por haber bebido, o tocado el agua? ¿O tal vez algún vecino o familiar?, ¿Y de algún animalito? (G4)

25. ¿Alguna vez ha percibido un olor extraño en el agua del río, o de la quebrada? ¿Y algún color o aspecto extraño tal vez?

26. ¿Alguna vez ha percibido un olor extraño en el agua que llega a su grifo o canal? ¿Y algún color o aspecto extraño tal vez? (G2)

27. En el caso de haberlos percibido ¿Cuáles podrían ser las causas de dichos olores o aspecto extraño del agua? (G4)

28. ¿A dónde van a parar las aguas de desagüe de su hogar? ¿tanto los servicios higiénicos, o cualquier tipo de desfogue o salida de agua de desecho? (G3)

29. ¿Sabes por qué las montañas, y especialmente las partes más altas, son importantes para que llegue el agua a los ríos? (G2)

30. ¿Qué recomendaciones daría usted, o que cree usted que debería cambiar o mejorar para que su comunidad tenga un servicio de agua suficiente y de calidad siempre? ¿Cómo cree que será el futuro con respecto al recurso del agua? ¿Qué debemos hacer o esperar? (G4)
